# Crystal structure of 1-(2,4-di­methyl­phen­yl)urea

**DOI:** 10.1107/S2056989014027431

**Published:** 2015-01-01

**Authors:** L. Jayalakshmi, C. Ramalingan, B. Sridhar, S. Selvanayagam

**Affiliations:** a Department of Chemistry, Kalasalingam University, Krishnankoil 626 126, India; b Laboratory of X-ray Crystallography, Indian Institute of Chemical Technology, Hyderabad 500 067, India; c Department of Physics & International Research Centre, Kalasalingam University, Krishnankoil 626 126, India

**Keywords:** crystal structure, urea, urea derivatives, hydrogen bonding

## Abstract

In the title urea derivative, C_9_H_12_N_2_O, the dihedral angle between the benzene ring and the mean plane of the urea group, N—C(=O)—N, is 86.6 (1)°. In the crystal, the urea O atom is involved in three N—H⋯O hydrogen bonds. Mol­ecules are linked *via* pairs of N—H⋯O hydrogen bonds, forming inversion dimers with an *R* ^2^_2_(8) ring motif. The dimers are linked by further N—H⋯O hydrogen bonds, forming two-dimensional networks lying parallel to (100).

## Related literature   

For general background to urea derivatives and their biological applications and properties, see: Ramalingan & Kwak (2008 ▸); Ramalingan *et al.* (2010 ▸); Yang *et al.* (2013 ▸); Safari & Gandomi-Ravandi (2014 ▸); Suzuki *et al.* (2013 ▸); Boulahjar *et al.* (2012 ▸); Zhang *et al.* (2014 ▸)
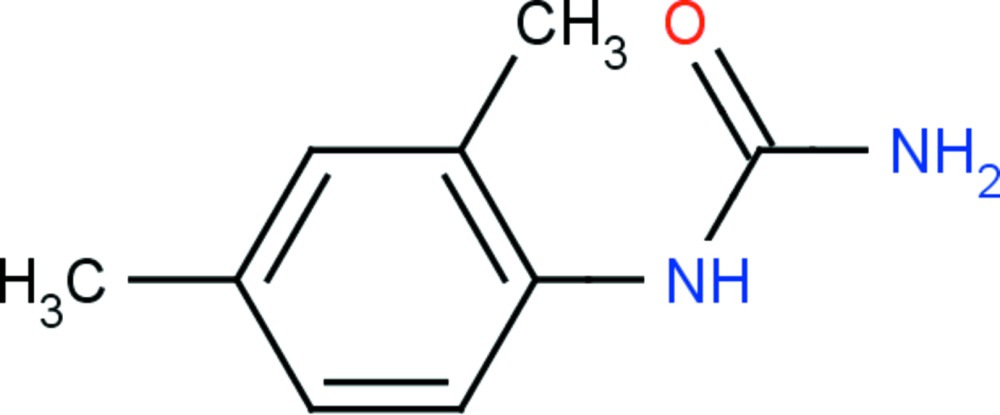


## Experimental   

### Crystal data  

C_9_H_12_N_2_O
*M* *_r_* = 164.21
Monoclinic, 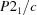

*a* = 14.631 (4) Å
*b* = 7.0633 (19) Å
*c* = 8.786 (2) Å
β = 93.530 (4)°
*V* = 906.2 (4) Å^3^
*Z* = 4
Mo *K*α radiation
μ = 0.08 mm^−1^
*T* = 292 K
0.20 × 0.18 × 0.16 mm

### Data collection  

Bruker SMART APEX CCD area-detector diffractometer
8026 measured reflections
1556 independent reflections
1284 reflections with *I* > 2σ(*I*)
*R*_int_ = 0.028

### Refinement  

*R*[*F* ^2^ > 2σ(*F* ^2^)] = 0.100
*wR*(*F* ^2^) = 0.349
*S* = 1.59
1556 reflections
119 parameters
2 restraints
H atoms treated by a mixture of independent and constrained refinement
Δρ_max_ = 0.87 e Å^−3^
Δρ_min_ = −0.32 e Å^−3^

### 

Data collection: *SMART* (Bruker, 2001 ▸); cell refinement: *SAINT* (Bruker, 2001 ▸); data reduction: *SAINT*; program(s) used to solve structure: *SHELXS97* (Sheldrick, 2008 ▸); program(s) used to refine structure: *SHELXL2013* (Sheldrick, 2008 ▸); molecular graphics: *ORTEP-3 for Windows* (Farrugia, 2012 ▸) and *PLATON* (Spek, 2009 ▸); software used to prepare material for publication: *SHELXL2013* and *PLATON* (Spek, 2009 ▸).

## Supplementary Material

Crystal structure: contains datablock(s) I, global. DOI: 10.1107/S2056989014027431/su5043sup1.cif

Structure factors: contains datablock(s) I. DOI: 10.1107/S2056989014027431/su5043Isup2.hkl

Supporting information file. DOI: 10.1107/S2056989014027431/su5043Isup3.cml

**Fig. 1.**
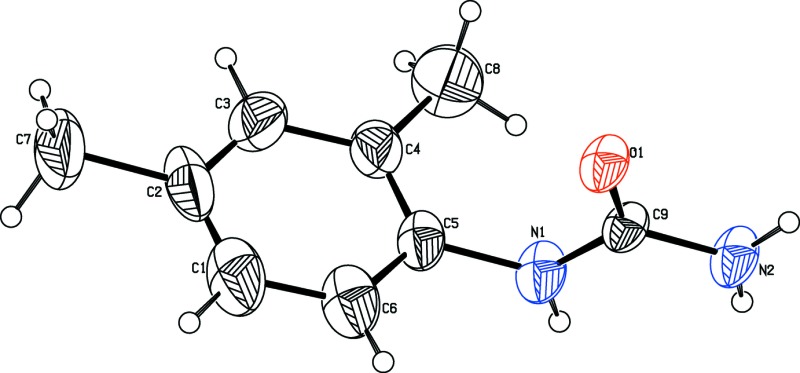
The molecular structure of the title compound, with atom labelling. Displacement ellipsoids are drawn at the 30% probability level.

**Fig. 2.**
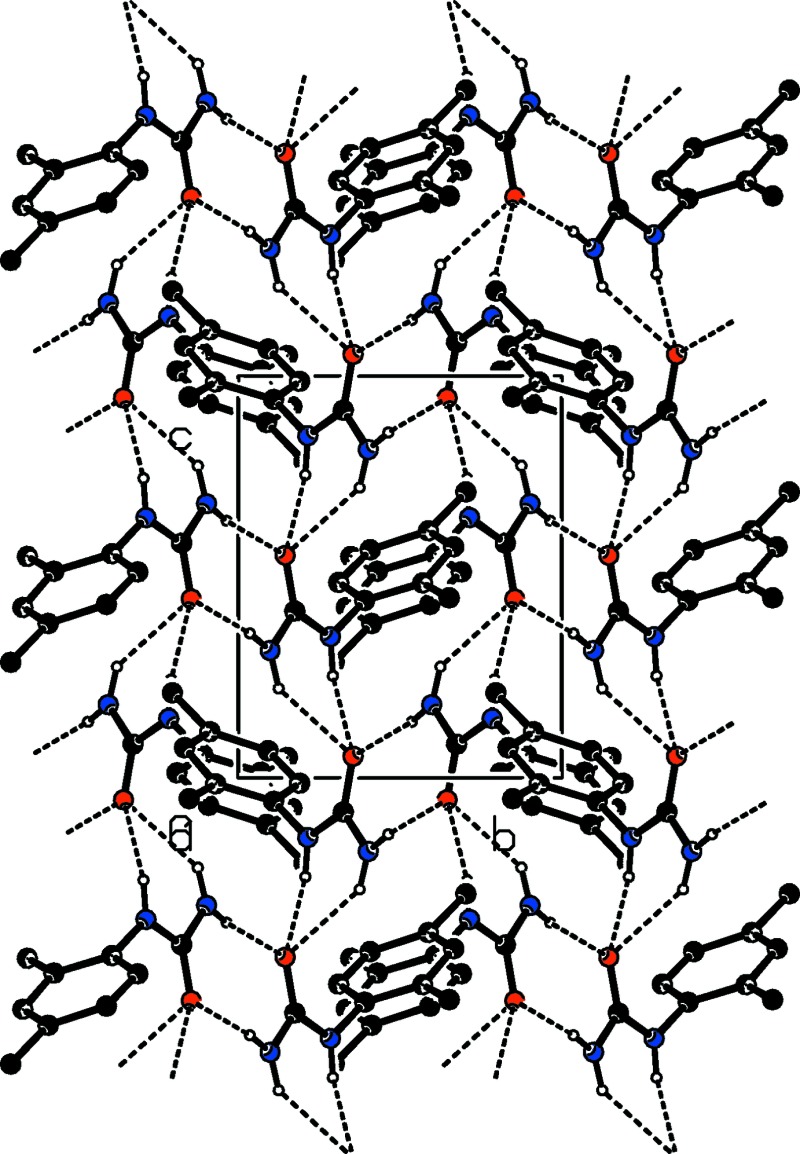
A projection of the crystal packing of the title compound, along the a axis. Hydrogen bonds are shown as dashed lines (see Table 1 for details; H atoms not involved in hydrogen bonding have been omitted for clarity).

. DOI: 10.1107/S2056989014027431/su5043fig1.tif
The mol­ecular structure of the title compound, with atom labelling. Displacement ellipsoids are drawn at the 30% probability level.

a . DOI: 10.1107/S2056989014027431/su5043fig2.tif
A projection of the crystal packing of the title compound, along the *a* axis. Hydrogen bonds are shown as dashed lines (see Table 1 for details; H atoms not involved in hydrogen bonding have been omitted for clarity).

CCDC reference: 1039538

Additional supporting information:  crystallographic information; 3D view; checkCIF report

## Figures and Tables

**Table 1:** Hydrogen-bond geometry (, )

*D*H*A*	*D*H	H*A*	*D* *A*	*D*H*A*
N1H1O1^i^	0.86	2.23	2.941(3)	140
N2H2*A*O1^i^	0.86(1)	2.24(2)	2.985(3)	145(3)
N2H2*B*O1^ii^	0.86(1)	2.12(1)	2.977(3)	173(4)

Symmetry codes: (i) 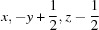; (ii) 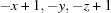.

## supplementary crystallographic information

### S1. Comment

Urea and its derivatives are important key starting materials for the construction of biologically important heterocycles (Ramalingan & Kwak, 2008; Ramalingan *et al.*, 2010; Yang *et al.*, 2013; Safari & Gandomi-Ravandi, 2014). They display various biological activities viz. antibacterial (Suzuki *et al.*, 2013), antiproliferative and antitumor (Boulahjar *et al.*, 2012), and HIV-1 integrase (Zhang *et al.*, 2014). As a vital reactant and intermediate for the construction of heterocyclic chemical entities of biological importance, the title compound has been synthesized and single crystals were grown by slow evaporation in ethanol.

The single crystal X-ray analysis confirmed the molecular structure, as illustrated in Fig. 1. Methyl carbon atoms, C7 and C8, deviate by -0.000 (1) and -0.040 (1) Å, respectively, from the attached benzene ring. The dihedral angle between benzene ring and the mean plane through the urea atoms (N1/C9/O1/N2) is 86.6 (1)°.

In the crystal, three strong N—H···O hydrogen bonds stabilize the molecular packing (Fig. 2 and Table 1). Molecules are linked via pairs of N-H···O hydrogen bonds forming inversion dimers with an R^2^_2_(8) ring motif. The dimers are linked by further N-H···O hydrogen bonds forming two-dimensional networks lying parallel to (100); see Table 1 and Fig. 2.

### S2. Experimental

To a solution of 2,6-dimethylaniline (0.1 mol) in glacial acetic acid (30 ml), was added distilled water (70 ml). Sodium cyanate (0.1 mol) in medium-hot water (50 ml) was then added in a slow manner with constant stirring. The resulted solution was allowed to stand for 60 min. and then cooled in ice. It was then filtered using a Buchner funnel and the solid obtained was dried using high-vacuum. Single crystals of the title compound were obtained by slow evaporation of a solution in ethanol at room temperature.

### S3. Refinement

Atoms H2A and H2B were located from a difference Fourier map and freely refined. The remaining H atoms were positioned geometrically and allowed to ride on their parent atoms, with N—H = 0.86 Å and C—H = 0.93-0.96 Å and with U_iso_(H) = 1.5U_eq_(C) for methyl H atoms and = 1.2U_eq_ (N,C) for other H atoms.

### Crystal data

**Table:**

C_9_H_12_N_2_O	*F*(000) = 352
*M**_r_* = 164.21	*D*_x_ = 1.204 Mg m^−^^3^
Monoclinic, *P*2_1_/*c*	Mo *K*α radiation, λ = 0.71073 Å
*a* = 14.631 (4) Å	Cell parameters from 6568 reflections
*b* = 7.0633 (19) Å	θ = 2.8–24.6°
*c* = 8.786 (2) Å	µ = 0.08 mm^−^^1^
β = 93.530 (4)°	*T* = 292 K
*V* = 906.2 (4) Å^3^	Block, colourless
*Z* = 4	0.20 × 0.18 × 0.16 mm

### Data collection

**Table:**

Bruker SMART APEX CCD area-detector diffractometer	*R*_int_ = 0.028
Radiation source: fine-focus sealed tube	θ_max_ = 25.0°, θ_min_ = 2.8°
ω scans	*h* = −17→17
8026 measured reflections	*k* = −8→8
1556 independent reflections	*l* = −10→10
1284 reflections with *I* > 2σ(*I*)	

### Refinement

**Table:**

Refinement on *F*^2^	2 restraints
Least-squares matrix: full	Hydrogen site location: mixed
*R*[*F*^2^ > 2σ(*F*^2^)] = 0.100	H atoms treated by a mixture of independent and constrained refinement
*wR*(*F*^2^) = 0.349	*w* = 1/[σ^2^(*F*_o_^2^) + (0.2*P*)^2^] where *P* = (*F*_o_^2^ + 2*F*_c_^2^)/3
*S* = 1.59	(Δ/σ)_max_ = 0.001
1556 reflections	Δρ_max_ = 0.87 e Å^−^^3^
119 parameters	Δρ_min_ = −0.32 e Å^−^^3^

### Special details

**Table:**

Geometry. All esds (except the esd in the dihedral angle between two l.s. planes) are estimated using the full covariance matrix. The cell esds are taken into account individually in the estimation of esds in distances, angles and torsion angles; correlations between esds in cell parameters are only used when they are defined by crystal symmetry. An approximate (isotropic) treatment of cell esds is used for estimating esds involving l.s. planes.

### Fractional atomic coordinates and isotropic or equivalent isotropic displacement parameters (Å^2^)

**Table:**

	*x*	*y*	*z*	*U*_iso_*/*U*_eq_	
O1	0.40043 (14)	0.1453 (3)	0.55423 (18)	0.0634 (8)	
N1	0.32526 (19)	0.2851 (4)	0.3520 (3)	0.0709 (10)	
H1	0.3186	0.2927	0.2543	0.085*	
N2	0.4476 (2)	0.0982 (4)	0.3174 (3)	0.0682 (9)	
H2A	0.437 (2)	0.125 (4)	0.2228 (15)	0.060 (8)*	
H2B	0.4934 (19)	0.036 (5)	0.360 (4)	0.089 (11)*	
C1	0.1253 (4)	0.4077 (12)	0.5679 (5)	0.1144 (17)	
H1A	0.0700	0.3530	0.5912	0.137*	
C2	0.1467 (3)	0.5877 (12)	0.6154 (4)	0.122 (2)	
C3	0.2259 (4)	0.6656 (8)	0.5753 (5)	0.1049 (17)	
H3	0.2404	0.7876	0.6085	0.126*	
C4	0.2873 (2)	0.5700 (6)	0.4854 (4)	0.0794 (11)	
C5	0.2652 (2)	0.3904 (5)	0.4421 (3)	0.0666 (10)	
C6	0.1852 (3)	0.3106 (8)	0.4870 (4)	0.0921 (13)	
H6	0.1721	0.1855	0.4606	0.111*	
C7	0.0822 (4)	0.7033 (13)	0.7118 (7)	0.178 (4)	
H7A	0.0806	0.6477	0.8114	0.267*	
H7B	0.0216	0.7030	0.6630	0.267*	
H7C	0.1040	0.8312	0.7212	0.267*	
C8	0.3708 (4)	0.6517 (7)	0.4485 (7)	0.1135 (16)	
H8A	0.3942	0.5853	0.3638	0.170*	
H8B	0.4142	0.6433	0.5348	0.170*	
H8C	0.3612	0.7822	0.4217	0.170*	
C9	0.39175 (19)	0.1749 (4)	0.4154 (3)	0.0519 (8)	

### Atomic displacement parameters (Å^2^)

**Table:**

	*U*^11^	*U*^22^	*U*^33^	*U*^12^	*U*^13^	*U*^23^
O1	0.0806 (14)	0.0787 (15)	0.0316 (11)	0.0198 (9)	0.0094 (9)	0.0014 (7)
N1	0.0824 (18)	0.0971 (19)	0.0338 (12)	0.0270 (14)	0.0098 (11)	0.0074 (11)
N2	0.0857 (18)	0.0849 (18)	0.0353 (14)	0.0251 (13)	0.0137 (11)	0.0013 (10)
C1	0.090 (3)	0.193 (5)	0.063 (2)	0.031 (3)	0.025 (2)	0.007 (3)
C2	0.068 (2)	0.245 (7)	0.053 (2)	0.063 (3)	0.0003 (17)	−0.015 (3)
C3	0.104 (3)	0.123 (3)	0.084 (3)	0.036 (3)	−0.019 (3)	−0.036 (2)
C4	0.0685 (19)	0.105 (3)	0.064 (2)	0.0184 (17)	−0.0010 (15)	−0.0088 (16)
C5	0.0682 (19)	0.090 (2)	0.0417 (16)	0.0245 (15)	0.0069 (13)	0.0057 (13)
C6	0.086 (2)	0.128 (3)	0.064 (2)	0.018 (2)	0.0218 (17)	0.0214 (19)
C7	0.106 (4)	0.316 (9)	0.112 (4)	0.091 (5)	−0.002 (3)	−0.091 (5)
C8	0.112 (3)	0.101 (3)	0.129 (4)	−0.017 (3)	0.024 (3)	0.004 (3)
C9	0.0652 (16)	0.0572 (15)	0.0342 (14)	0.0069 (11)	0.0094 (11)	−0.0013 (9)

### Geometric parameters (Å, º)

**Table:**

O1—C9	1.236 (3)	C3—C4	1.406 (6)
N1—C9	1.340 (4)	C3—H3	0.9300
N1—C5	1.428 (4)	C4—C5	1.358 (6)
N1—H1	0.8600	C4—C8	1.407 (7)
N2—C9	1.337 (4)	C5—C6	1.377 (6)
N2—H2A	0.857 (10)	C6—H6	0.9300
N2—H2B	0.863 (10)	C7—H7A	0.9600
C1—C6	1.351 (7)	C7—H7B	0.9600
C1—C2	1.368 (10)	C7—H7C	0.9600
C1—H1A	0.9300	C8—H8A	0.9600
C2—C3	1.348 (9)	C8—H8B	0.9600
C2—C7	1.541 (6)	C8—H8C	0.9600
			
C9—N1—C5	121.9 (2)	C6—C5—N1	120.5 (4)
C9—N1—H1	119.1	C1—C6—C5	122.2 (6)
C5—N1—H1	119.1	C1—C6—H6	118.9
C9—N2—H2A	117 (2)	C5—C6—H6	118.9
C9—N2—H2B	115 (3)	C2—C7—H7A	109.5
H2A—N2—H2B	128 (4)	C2—C7—H7B	109.5
C6—C1—C2	119.2 (6)	H7A—C7—H7B	109.5
C6—C1—H1A	120.4	C2—C7—H7C	109.5
C2—C1—H1A	120.4	H7A—C7—H7C	109.5
C3—C2—C1	119.0 (4)	H7B—C7—H7C	109.5
C3—C2—C7	119.5 (7)	C4—C8—H8A	109.5
C1—C2—C7	121.5 (6)	C4—C8—H8B	109.5
C2—C3—C4	122.7 (5)	H8A—C8—H8B	109.5
C2—C3—H3	118.7	C4—C8—H8C	109.5
C4—C3—H3	118.7	H8A—C8—H8C	109.5
C5—C4—C3	117.1 (4)	H8B—C8—H8C	109.5
C5—C4—C8	121.0 (4)	O1—C9—N2	122.5 (2)
C3—C4—C8	121.8 (4)	O1—C9—N1	122.4 (2)
C4—C5—C6	119.7 (3)	N2—C9—N1	115.1 (2)
C4—C5—N1	119.8 (3)		
			
C6—C1—C2—C3	2.3 (7)	C8—C4—C5—N1	−2.8 (5)
C6—C1—C2—C7	−178.3 (4)	C9—N1—C5—C4	90.7 (4)
C1—C2—C3—C4	0.5 (7)	C9—N1—C5—C6	−88.6 (4)
C7—C2—C3—C4	−178.9 (4)	C2—C1—C6—C5	−4.1 (7)
C2—C3—C4—C5	−1.6 (6)	C4—C5—C6—C1	2.9 (6)
C2—C3—C4—C8	−178.1 (5)	N1—C5—C6—C1	−177.8 (3)
C3—C4—C5—C6	−0.1 (5)	C5—N1—C9—O1	6.1 (5)
C8—C4—C5—C6	176.5 (4)	C5—N1—C9—N2	−174.4 (3)
C3—C4—C5—N1	−179.4 (3)		

### Hydrogen-bond geometry (Å, º)

**Table:**

*D*—H···*A*	*D*—H	H···*A*	*D*···*A*	*D*—H···*A*
N1—H1···O1^i^	0.86	2.23	2.941 (3)	140
N2—H2*A*···O1^i^	0.86 (1)	2.24 (2)	2.985 (3)	145 (3)
N2—H2*B*···O1^ii^	0.86 (1)	2.12 (1)	2.977 (3)	173 (4)

Symmetry codes: (i) *x*, −*y*+1/2, *z*−1/2; (ii) −*x*+1, −*y*, −*z*+1.
