# Supplementary figures and images for: Crystal structure of 1-(2,4-di­methyl­phen­yl)urea
Source: Acta Crystallogr E Crystallogr Commun. 2015 Jan 1;71(Pt 1):o60–1. doi: 10.1107/S2056989014027431 (PMC4331890; doi:10.1107/S2056989014027431)

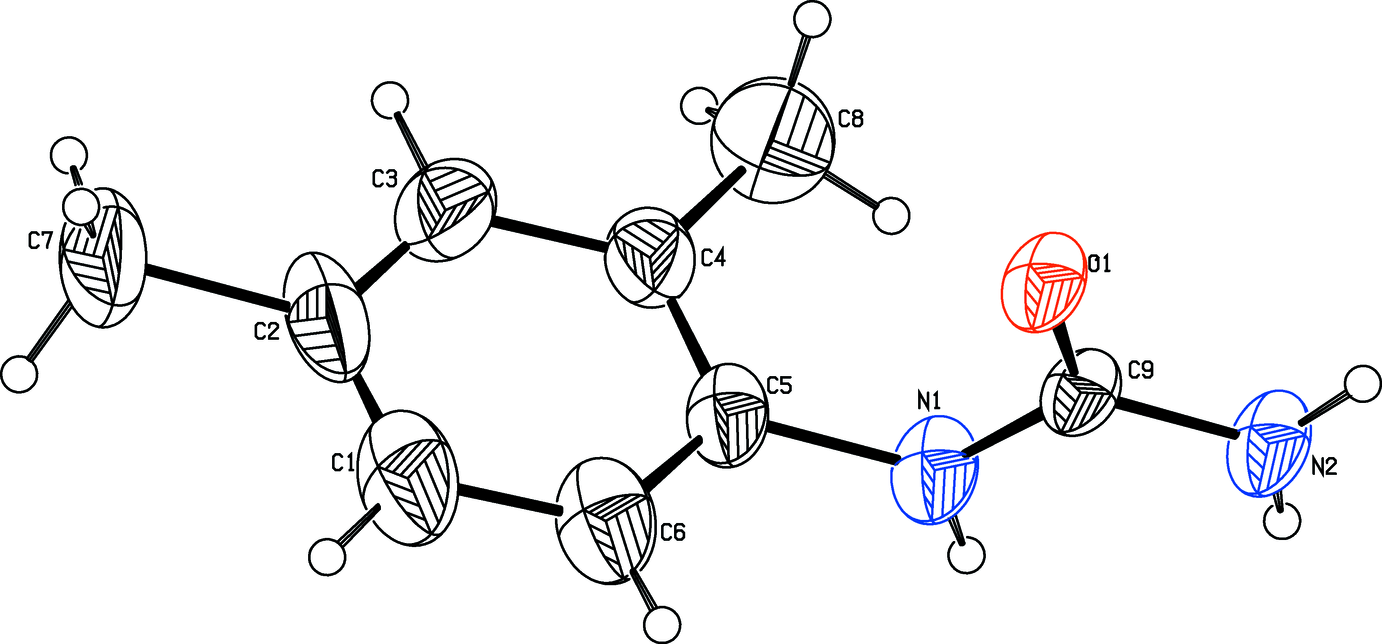

Supplement: Supplementary file 4 [file e-71-00o60-fig1.tif]

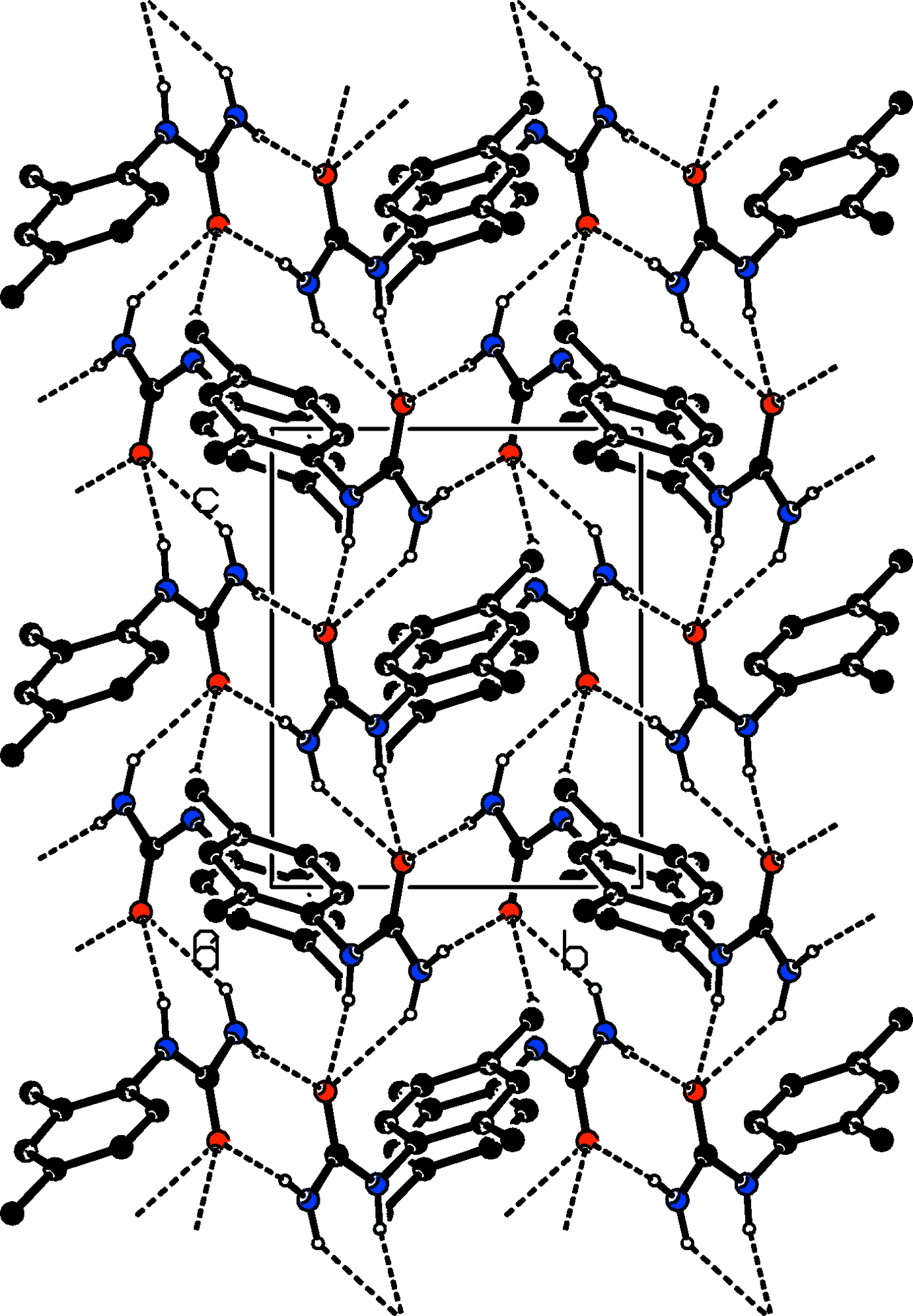

Supplement: Supplementary file 5 [file e-71-00o60-fig2.tif]
